# Bilateral dynamic cerebral autoregulation assessment during endovascular treatment in large‐vessel occlusion stroke

**DOI:** 10.1113/EP093024

**Published:** 2025-11-20

**Authors:** Adam Vittrup Heiberg, Troels Gil Lukassen, Thomas Clement Truelsen, Henrik Gutte Borgwardt, Goetz Benndorf, Christine Sølling, Henrik Winther Schytz, Klaus Hansen, Kirsten Møller, Helle Klingenberg Iversen

**Affiliations:** ^1^ Department of Neurology Copenhagen University Hospital – Rigshospitalet Glostrup Denmark; ^2^ Department of Clinical Medicine University of Copenhagen Copenhagen N Denmark; ^3^ Department of Diagnostic Radiology Copenhagen University Hospital – Rigshospitalet Copenhagen East Denmark; ^4^ Department of Diagnostic Radiology Baylor College of Medicine Houston Texas USA; ^5^ Department of Neuroanaesthesiology Copenhagen University Hospital – Rigshospitalet Copenhagen East Denmark

**Keywords:** acute ischaemic stroke, cerebral circulation, dynamic cerebral autoregulation, endovascular treatment, large‐vessel occlusion, near‐infrared spectroscopy, thrombectomy, transfer function analysis

## Abstract

Recanalization by endovascular treatment (EVT) is effective in acute ischaemic stroke caused by large‐vessel occlusion. Better understanding of the pathophysiology could possibly identify targets for improving peri‐procedural management and thereby patient outcome. Dynamic cerebral autoregulation (dCA), which maintains cerebral blood flow despite changes in arterial blood pressure (ABP), is reportedly impaired after EVT. Blood pressure thresholds after EVT have previously been individualized by accounting for dCA, which could improve outcome. The conventional method to estimate dCA requires transcranial Doppler, which is difficult to use during EVT. Instead, we investigated dCA during EVT by near‐infrared spectroscopy (NIRS) which is more feasible. NIRS and ABP were measured continuously before recanalization, immediately after recanalization, and after general anaesthesia termination for subsequent transfer function analysis yielding the dCA measure of phase shift (0.07−0.2 Hz). Phase shift did not differ between the ischaemic and contralateral hemisphere but the sensitivity to end‐tidal CO_2_ was increased in the ischaemic hemisphere immediately after recanalization. Phase shift over time interacted with 90‐day functional outcome including independence and mortality. Hence, patients with good long‐term outcome showed increased phase shift during and after EVT, while phase shift decreased in poor outcome patients. In conclusion, dCA did not differ between hemispheres during EVT but was more sensitive to end‐tidal CO_2_ in the ischaemic compared to the contralateral hemisphere and dCA evolved differently in patients with good and poor outcome. Our findings of individual dCA differences during EVT suggest benefit of individualized blood pressure management, which should be addressed in future studies.

## Abstract (highlights)

- **What is the central question of this study?**
  Dynamic cerebral autoregulation (dCA), which maintains blood flow constant despite changes in blood pressure, is impaired in acute ischaemic stroke patients after endovascular treatment (EVT) but has not been examined during EVT.
- **What is the main finding and its importance?**
  Assessment of dCA during EVT was feasible analysing phase shift (PS) between near infrared spectroscopy and blood pressure. PS sensitivity to end‐tidal CO_2_ was increased in the ischaemic hemisphere. PS increased during EVT for patients with good long‐term outcome and vice versa. The findings could indicate a benefit of individualized intraoperative management.

## 1 INTRODUCTION

Acute ischaemic stroke due to large‐vessel occlusion forms a substantial proportion of all strokes and has the worst prognosis (Malhotra et al., 2017). However, recanalization by endovascular treatment (EVT) markedly improves the outcome of stroke patients whether performed in the anterior or posterior circulation (Baik et al., 2023; Goyal et al., 2016; Nogueira et al., 2018). While highly effective, EVT can still be improved by limiting procedure‐related complications and improved understanding of mechanisms underlying futile recanalization. Cerebral autoregulation impairment has been proposed as one of the reasons for futile recanalization (Nogueira et al., 2022; Wang & Xiong, 2023) and has successfully been applied to individualize blood pressure threshold after EVT (Petersen et al., 2020).

Cerebral autoregulation is the complex mechanism of maintaining a suitable blood flow despite alterations in cerebral perfusion pressure (Paulson et al., 1990). When rapid changes in perfusion pressure occur, cerebral blood flow is maintained by dynamic cerebral autoregulation (dCA)(Claassen et al., 2021). Conventional dCA examination relies on simultaneous blood pressure monitoring and transcranial Doppler sonography (TCD) with analysis of low‐frequency oscillations (LFO, approximately 0.1 Hz) observed in both systemic (i.e., arterial blood pressure, ABP) and cerebral circulation (Andersen et al., 2018; Obrig et al., 2000; Reinhard et al., 2006). The most standardized method is transfer function analysis (TFA), which quantifies input (ABP) and output (blood flow velocity assessment by TCD in the middle cerebral artery, *V*_MCA_) in low‐frequency spectrums (Liu et al., 2020; Panerai et al., 2023). TFA estimates dCA by the attenuation (gain or amplitude ratio) and temporal displacement (phase shift) of LFOs. Thus, impaired dCA is associated with increased gain and reduced phase shift; conversely, reduced gain and increased phase shift signify enhanced dCA.

TCD requires expertise and setup time, which reduce the inter‐rater variability substantially (Bhuiyan et al., 2012; Nedelmann et al., 2009). Continuous TCD during EVT requires equipment that would likely interfere with the procedure of digital subtraction angiography. In contrast, near‐infrared spectroscopy (NIRS) can be applied throughout the procedure as it requires almost no setup time and is compatible with digital subtraction angiography. NIRS is an optical modality that measures continuous and dynamic changes in cortical haemoglobin concentrations as a marker of regional cerebral blood flow (Andersen et al., 2018; Ferrari & Quaresima, 2012). Hence, NIRS can replace TCD in the conventional dCA examination setup (Obrig et al., 2000; Phillip et al., 2012; Reinhard et al., 2006).

Studies of dCA in large‐vessel occlusion have consistently reported impaired autoregulation in the ischaemic hemisphere (Intharakham et al., 2019; Nogueira et al., 2022) with ischaemic or penumbral tissue vulnerable to hypo‐ and hyperperfusion. Further, dCA impairment in the affected hemisphere has been associated to both short‐term complications (Castro, Azevedo et al., 2017) and long‐term outcome (Nogueira et al., 2022). We previously examined interhemispheric dCA during EVT, finding that the strength of autoregulation expressed by interhemispheric gain was predictive of 90‐day outcome (Heiberg et al., 2025). Moreover, interhemispheric phase shift already increased during EVT in patients with milder symptom severity. The ischaemic and the contralateral hemispheres were well‐synchronized, indicating no or similar phase shift changes bilaterally (Heiberg et al., 2025). Finally, conventional dCA examinations (ABP‐*V*_MCA_) of large‐vessel occlusion showed impairment after EVT in the contralateral hemisphere in some (Meyer et al., 2020; Tian et al., 2020) but not all studies (Salinet et al., 2019).

Thus, the aim of the current study was to explore dCA during EVT in both the ischaemic and the contralateral hemisphere using ABP and NIRS for TFA.

## 2 METHODS

### 2.1 Ethical approval

The study was approved by the Scientific Ethics Committees for the Capital Region of Denmark (H‐18028704) and performed in accordance with the World Medical Association *Declaration of Helsinki* (ClinicalTrials.gov: NCT03738644). Informed consent was given in writing by all participants or their proxy. The study adhered to the policies of *Experimental Physiology* regarding human experiments and complied with STrengthening the Reporting of OBservational studies in Epidemiology (STROBE) guidelines for observational studies (von Elm et al., 2007).

### 2.2 Enrolment

**FIGURE 1:**
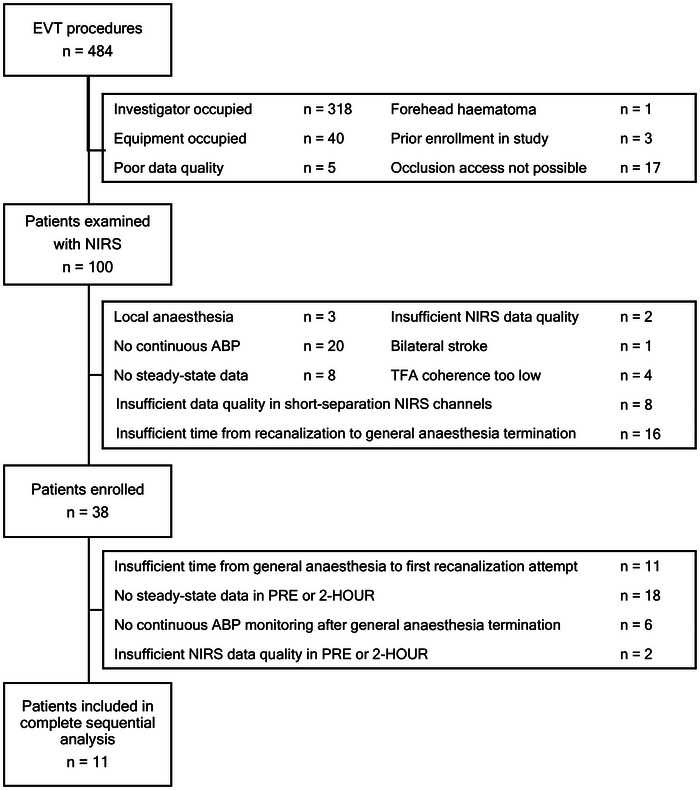
Enrolment flowchart including exclusion criteria.

Acute ischaemic stroke patients with large‐vessel occlusion admitted at a single comprehensive stroke centre, Rigshospitalet, and receiving EVT were screened for eligibility between November 2018 and November 2020. Rigshospitalet covers Eastern Denmark with a population of approximately 2.7 million people. The enrolment process is shown in Figure 1 including exclusion criteria.

### 2.3 Treatment and monitoring

Patients were diagnosed and treated as per standard‐of‐care. Large‐vessel occlusion was confirmed prior to acute EVT, which was performed under general anaesthesia (GA) in all enrolled patients without intraarterial vasodilators. Alberta Stroke Program Early Computed Tomography Score (ASPECTS) (Barber et al., 2000) or Posterior circulation‐ASPECTS (PC‐ASPECTS)(Puetz et al., 2008) were assessed by staff neuroradiologists assisted by RAPID AI software (RRID:SCR_027023, iSchemaView, Menlo Park, CA, USA) and dichotomized to favourable (ASPECTS/PC‐ASPECTS ≥8) or unfavourable (ASPECTS/PC‐ASPECTS <8). Patients had symptom severity reassessed using the National Institutes of Health Stroke Scale (NIHSS) upon arrival at the angio‐suite immediately prior to EVT. Continuous standard‐of‐care monitoring including invasive arterial blood pressure (Philips IntelliVue, Philips Medical Systems, Eindhoven, The Netherlands) was performed from arrival to the angio suite until 2 h after EVT and exported by VSCapture (RRID:SCR_027024) (Karippacheril & Ho, 2013) with additional NIRS monitoring (described below). Recanalization grading was determined by the treating interventional neuroradiologist (modified Treatment In Cerebral Infarction, mTICI (Zaidat et al., 2013): grade 0–2a ranging from unsuccessful reperfusion to reperfusion in less than half of the occluded artery territory; grade 2b–3 defined as successful reperfusion in more than half of the occluded artery territory or complete reperfusion). Patients were extubated as soon as possible after EVT. Dual‐energy computed tomography or magnetic resonance imaging was performed 24 h after EVT to assess infarction size (ASPECTS/PC‐ASPECTS) and detect complications including intracranial haemorrhage and embolization to new vascular territory. Patients underwent stroke work‐up as per standard‐of‐care. A stroke aetiology classification was performed using the Causative Classification System for Ischemic Stroke (Ay et al., 2007). Further details concerning neuroimaging, anaesthesia and the NIRS equipment can be found elsewhere (Heiberg et al., 2025).

### 2.4 Clinical follow‐up

Clinical follow‐up was performed 24 h (±6 h from end of endovascular reperfusion efforts) and 90 days (±14 days) after EVT, which included NIHSS assessment and functional outcome by the Modified Rankin Scale (mRS) and independence (defined as mRS of 0–2). Re‐hospitalizations, new vascular events (i.e. recurrent stroke, myocardial infarction, or surgery for peripheral artery disease) and all‐cause mortality were recorded at 90 days using the electronic health record system. Patients, who did not attend in‐person 90‐day follow‐up, were interviewed over telephone or by proxy.

### 2.5 NIRS examination

Patients underwent dynamic cortical haemoglobin concentration measurement with NIRS using a continuous wave‐system (Octamon, Artinis Medical Systems, Elst, the Netherlands) with three long‐distance channels (35 mm) per hemisphere (Heiberg et al., 2025). Channels covered the prefrontal cortex both in the border zones areas (two channels) supplied by the middle cerebral artery (MCA) and in the anterior cerebral arteries (ACA) and in the exclusive ACA territory (one channel) (Koenig et al., 2021). Extracerebral tissue was examined by one short‐distance channel per side (10 mm).

### 2.6 Time segment selection

Steady state time segments of 5 min were selected as recommended by the Cerebrovascular Research Network (Panerai et al., 2023). Therefore, we did not include any data segments within 5 min of inducing GA and 2 min after the occurrence of substantial changes in anaesthetics, opioids or vasopressors (Llwyd et al., 2022). Data segments with excessive noise were also excluded. We allowed a 10% variation of recorded vitals within the time segment.

We specified the first time segment (PRE) between the induction of GA and prior to any revascularization attempts. The second time segment (POST) was identified immediately after final reperfusion status was achieved which included abandonment in case of unsuccessful recanalization attempts and before GA termination. The third time segment was defined between GA termination and as late as possible (up to 2 h from recanalization) mainly based on avoiding major motion artifacts. Due to missing steady state before recanalization attempts and setup time of invasive blood pressure measurement, the most available time segment during EVT was POST by a wide margin, and patients without valid POST segment data were excluded from analysis. A subset of patients with all available time segments (complete sequential subset) were included for analysis over time.

### 2.7 Data analysis

Analysis was performed as recommended in TFA guidelines from The Cerebrovascular Research Network (RRID:SCR_027022) (Panerai et al., 2023). Raw NIRS data were converted to oxygenated haemoglobin (Oxy‐Hb) by the modified Beer–Lambert law (Homer2, RRID:SCR_009586). We chose Oxy‐Hb over other NIRS parameters as it has superior data quality and correlates better to flow changes in the arterial and arteriolar compartment (Gomez et al., 2022; Polinder‐Bos et al., 2020; Reinhard et al., 2006) where autoregulation occurs. ABP and Oxy‐Hb were corrected for noise by linear interpolation before beat‐to‐beat averaging and resampling at 10 Hz. Input to TFA was ABP and output was Oxy‐Hb from both the ischaemic and contralateral hemisphere. The Welch’ method was applied which resulted in power spectral density, coherence, gain (amplitude ratio), normalized gain (accounting for different measurement ranges and units) and phase shift calculated in three frequency intervals: High‐frequency (HF, 0.2−0.5 Hz), low‐frequency (LF 0.07−0.2 Hz) and very low‐frequency (VLF, 0.02−0.07 Hz)(Panerai et al., 2023). All data analysis was performed in MATLAB (MathWorks, Natick, MA, USA; RRID:SCR_001622).

Gain is usually interpreted as a dCA quantification given by the damping ability of oscillations in ABP relative to oscillations in Oxy‐Hb. When gain equals 1, dCA is suspected to be fully impaired as ABP oscillations are transferred passively to the cerebral circulation. Vice versa, increasingly intact dCA would result in a progressively lower gain (<1). Phase shift denotes the temporal displacement between signals induced by dCA (Diehl et al., 1995). A decreasing phase shift between ABP and *V*_MCA_ is indicative of increasingly impaired dCA (Claassen et al., 2021). When dCA is intact, LFOs in *V*_MCA_ precede those in ABP but the relation is conventionally determined as the phase lead by *V*_MCA_, which is a positive number. Meanwhile, LFOs in OxyHb succeeds those in ABP in healthy subjects (Müller & Österreich, 2019; Phillip et al., 2012; Reinhard et al., 2006). In this paper we describe phase shift as negative numbers when LFOs precede ABP and positive numbers when they occur after ABP.

A common problem in TFA is phase wrap‐around of 360 degrees, which we did not observe. However, a minority of single channels did exhibit an accurate 180° move from the remaining channels. We suspect this phenomenon could be induced by low amplitudes in the LF and VLF ranges. Therefore, we corrected single channel phase shifts by 180° when other channels were consistent.

Data analysis and statistics were performed by the first author unblinded to outcome.

### 2.8 Statistics

All statistical analysis was conducted with R (R Foundation for Statistical Computing, Vienna, Austria; RRID:SCR_001905). Data are described by mean and standard deviation if normally distributed and by median and interquartile range (IQR) for non‐normally distributed data. Overall results from TFA were analysed from the side‐to‐side difference by appropriate paired tests. The complete sequential subset was analysed across time segments by multiple comparisons adjusted with false discovery rate as well as side‐to‐side differences at each time segment.

We used the lme4 package with internal convergence check to fit linear mixed‐effect models in with LF phase shift as outcome, subjects as random effect, and fixed effects comprising hemisphere and different patient characteristics (e.g. stroke aetiology, carotid or intracranial stenosis ≥50%, patient age, baseline NIHSS, average ABP, end‐tidal carbon dioxide ($ET_{CO_{2}}$), favourable ASPECTS/PC‐ASPECTS before EVT and occluded territory categorized to posterior circulation, anterior cerebral artery including internal carotid occlusions, and isolated middle cerebral artery occlusions), treatment (e.g. intravenous thrombolysis (IVT) administered before EVT, recanalization success, time from last‐known‐well to final recanalization status, immediate 2‐h NIHSS improvement) or 90‐day outcome (e.g. NIHSS, mRS, independence, mortality).

For analysis in the complete sequential subset, time segment was added as a fixed effect and interaction between time segment and the fixed effects mentioned above were assessed to determine different progressions over time between groups. Some fixed effects did not meet statistical requirements for sample size in the complete sequential subset and were excluded.

Estimates (β) are presented with 95% confidence intervals (CI). The level of significance was set at 5%.

## 3 RESULTS

We included 38 patients in the study of which 11 patients were eligible for complete sequential analysis. Baseline information including medical history is presented in Table 1 for all patients and comparative description of patients eligible and ineligible for the complete sequential subset. The complete sequential subset had significantly higher peripheral artery disease in their medical history.

**TABLE 1:** Baseline information and medical history.

	All patients (*n* = 38)	Complete sequential subset (*n* = 11)	Only POST (*n* = 27)	*P*
Age, mean (SD)	69.6 (13.9)	74.0 (11.0)	67.8 (14.8)	0.334
Female sex, *n* (%)	13 (34.2)	5 (45.5)	8 (29.6)	0.457
Caucasian ethnicity, *n* (%)	35 (92.1)	9 (81.8)	26 (96.3)	0.196
Right‐handed, *n* (%)	32 (84.2)	11 (100)	21 (77.8)	0.459
BMI, mean (SD)	26.1 (6.1)	25.5 (5.6)	26.4 (6.4)	0.446
Smoking, *n* (%)				
Never	12 (31.6)	3 (27.3)	9 (33.3)	1.000
Former	13 (34.2)	6 (54.5)	7 (25.9)	0.136
Current	13 34.2)	2 (18.2)	11 (40.7)	0.268
Alcohol consumption (units weekly), median	0.5 (0.0; 6.75)	0 (0; 8)	1.0 (0.0; 6.5)	0.959
Physical inactivity^a^, *n* (%)	11 (71.1)	2 (18.2)	9 (33.3)	0.452
Medical history, *n* (%)				
Prior ischaemic stroke	5 (13.2)	3 (27.3)	2 (7.4)	0.134
Prior TIA	2 (5.3)	0 (0)	2 (7.4)	1.000
Hypertension	24 (63.2)	9 (81.8)	15 (55.6)	0.160
Diabetes	7 (18.4)	2 (18.2)	5 (18.5)	1.000
Extracranial artery stenosis ≥ 50%	11 (28.9)	5 (45.5)	6 (22.2)	0.238
Ipsilateral	10 (26.3)	5 (45.5)	5 (18.5)	0.116
Contralateral	6 (15.8)	3 (27.3)	3 (11.1)	0.328
Intracranial artery stenosis ≥ 50%	7 (18.4)	3 (27.3)	4 (14.8)	0.390
Atrial fibrillation	17 (44.7)	6 (54.5)	11 (40.7)	0.491
Dyslipidaemia	36 (94.7)	9 (81.8)	27 (100)	0.078
Ischaemic heart disease	2 (5.3)	1 (9.1)	1 (3.7)	0.512
Valvular heart disease	6 (15.8)	1 (9.1)	5 (18.5)	1.000
Heart failure	4 (10.5)	0 (0)	4 (14.8)	0.557
Peripheral artery disease	3 (7.9)	3 (27.3)	0 (0.0)	0.017
Nephropathy	5 (13.2)	3 (27.3)	2 (7.4)	0.134
Venous thromboembolism	3 (7.9)	1 (9.1)	2 (7.4)	1.000
Disseminated cancer	2 (5.3)	2 (18.2)	0 (0)	0.078

^a^ Defined as less than 1 h weekly.

Abbreviations: BMI: body‐mass index; TIA: transient ischaemic attack.

Index stroke statistics including treatment and outcome are presented in Tables 2 and 3. The time from arterial puncture to reperfusion was numerically longer in the complete sequential subset, which is expected as all these patients had available steady‐state periods. However, the time from last‐known‐well to reperfusion was not different. Patients in the complete sequential subset had higher ASPECTS/PC‐ASPECTS before EVT and 24 h after EVT, which was not significantly associated with any clinical short‐term or long‐term outcome.

**TABLE 2:** Index stroke characteristics.

	All subjects (*n* = 38)	Complete sequential subset (*n* = 11)	Only POST (*n* = 27)	*P*
Ischaemic hemisphere, right side, *n* (%)	20 (52.6)	5 (45.5)	15 (55.6)	0.724
Onset, *n* (%)				
Wake‐up	7 (18.4)	1 (9.1)	6 (22.2)	0.648
Unwitnessed	7 (18.4)	3 (27.3)	4 (14.8)	0.390
Stroke aetiology, *n* (%)				
Large‐artery atherosclerosis	15 (39.5)	5 (45.5)	10 (37.0)	0.722
Cardio‐aortic embolism	14 (36.8)	3 (27.3)	11 (40.7)	0.488
Other causes (dissection)	3 (7.9)	1 (9.1)	2 (7.4)	1.000
Undetermined causes	6 (15.8)	2 (18.2)	4 (14.8)	1.000
Occluded artery, *n* (%)				
ICA	2 (5.3)	1 (9.1)	0 (0.0)	0.501
ICA‐top	5 (13.2)	2 (18.2)	3 (11.1)	0.615
ICA‐tandem	5 (13.2)	2 (18.2)	3 (11.1)	0.615
M1	23 (60.5)	7 (63.6)	16 (59.3)	1.000
M2	15 (39.5)	5 (45.5)	10 (37.0)	0.722
ACA	4 (10.5)	1 (9.1)	3 (11.1)	1.000
BA	2 (5.3)	1 (9.1)	1 (3.7)	0.501
PCA	3 (7.9)	0 (0.0)	3 (11.1)	0.542
ASPECTS/PC‐ASPECTS				
Before EVT, median (IQR)	8 (7, 10)	9 (8.5, 10)	8 (7, 10)	0.040
Favourable before EVT (≥8), *n* (%)	27 (71.1)	11 (100)	16 (59.3)	0.016
24‐h, median (IQR)	7 (5, 9)	8 (7, 9)	6 (4.5, 8)	0.034

Abbreviations: ACA, anterior cerebral artery; ASPECTS, Alberta Stroke Program Early CT Score; BA, basilar artery; EVT, endovascular treatment; ICA, internal carotid artery; M1, first segment of the middle cerebral artery; M2, second segment of the middle cerebral artery; mTICI, modified treatment in cerebral infarction; PCA, posterior cerebral artery; PC‐ASPECTS, posterior circulation ASPECTS.

**TABLE 3:** Index stroke treatment and outcome.

	All subjects (*n* = 38)	Complete sequential subset (*n* = 11)	Only POST (*n* = 27)	*P*
IVT, *n* (%)	19 (50.0)	5 (45.5)	14 (51.9)	1.000
Procedure, *n* (%)				
Aspiration only	11 (28.9)	2 (18.2)	9 (33.3)	0.452
Stent‐retrieving and aspiration	27 (71.1)	9 (81.8)	18 (66.7)	0.452
PTA	6 (15.8)	3 (27.3)	3 (11.1)	0.329
Carotid stenting	2 (5.3)	2 (18.2)	0 (0)	0.078
Anaesthetics				
Propofol				
*n* (%)	36 (94.7)	11 (100)	25 (92.6)	1.000
Max. dose (µg/min/Kg, range)	67 (50, 149)	67 (50, 149)	66 (51, 107)	0.559
Sevoflurane				
*n* (%)	2 (5.3)	0 (0)	2 (7.4)	1.000
Max. dose (MAC, range)	0.9 (0.8‐−1.0)	N/A	0.9 (0.8‐−1.0)	N/A
Vasopressor, *n* (%)				
Phenylephrine	22 (57.9)	7 (63.6)	15 (55.6)	0.729
Noradrenaline	3 (7.9)	0 (0)	3 (11.1)	0.542
Combination of phenylephrine and noradrenaline	13 (34.2)	4 (36.4)	9 (33.3)	1.000
Successful reperfusion (mTICI ≥ 2b), *n* (%)	32 (84.2)	10 (90.9)	22 (81.5)	0.650
Last‐known‐well to reperfusion in min^a^	299 (225, 527)	311 (260, 472)	287 (173, 709)	0.459
NIHSS, median (IQR)				
Before EVT	16.5 (9.5, 22)	14 (7.5, 16)	18 (12.5, 22.5)	0.142
2 h after EVT	11 (5.5, 13.5)	9 (4.5, 11.5)	12.5 (6, 14)	0.105
24 h after EVT	6.5 (4, 12)	6 (5, 9)	7 (3.5, 13)	0.508
90‐day	2.5 (0, 4)	1 (0, 4)	3 (0.5, 4.5)	0.898
90‐day mRS, median (IQR)	3 (2, 4)	2 (2, 4.5)	3 (2, 4)	0.844
90‐day independence^b^, *n* (%)	21 (55.3)	6 (45.5)	11 (40.7)	0.491
Vascular events^c^, *n* (%)	3 (7.9)	1 (9.1)	2 (7.4)	1.000
Complications, *n* (%)				
New territory embolization	5 (13.2)	1 (9.1)	4 (11.1)	1.000
Intracranial haemorrhage	4 (10.5)	2 (18.2)	2 (7.4)	0.564
Other complications^d^	2 (5.3)	0 (0)	2 (7.4)	1.000
All‐cause mortality, *n* (%)				
90‐day	6 (15.8)	2 (18.2)	4 (14.8)	1.000
1 year	7 (18.4)	2 (18.2)	5 (18.5)	1.000

^a^ In case of mTICI 0, defined as time of abandoning EVT efforts.

^b^ Intracerebral artery dissection or significant groin haematoma.

^c^ Modified Rankin Scale 0−2.

^d^ Defined as recurrent stroke, myocardial infarction, or surgery for peripheral artery disease at 90‐day FU.

Abbreviations: EVT: Endovascular treatment; IVT, intravenous thrombolysis; MAC, minimum alveolar concentration; mTICI, modified treatment in cerebral infarction; mTICI: Modified treatment in cerebral infarction; PTA, percutaneous transluminal angioplasty.

Results from the ABP‐OxyHb TFA along with examination time and vitals are presented in Table 4. We found no difference between the hemispheres of any TFA measures. Phase shift for both hemispheres was approximately 40 degrees in the LF range.

**TABLE 4:** Examination time, average vitals and TFA results immediately after recanalization.

Variable	Specification	Value	
Examination time from last‐known‐well (h)		5.4 (4.0, 8.9)	
HR (bpm, mean)		63.3 (12.0)	
$S_{pO_{2}}$ (%)		100 (99, 100)	
$ET_{CO_{2}}$ (kPA, mean)		4.59 (0.45)	
Mean ABP (mmHg)	Before EVT	110.3 (92.8, 118.8)^c^	
	During EVT^a^	80.6 (75.8, 87.2)^c^	
	After EVT^b^	93 (84.0, 101.6)^c^	
ABP PSD (mmHg^2^/Hz)	HF	1.37 (0.91, 4.18)	
	LF	1.07 (0.43, 1.83)	
	VLF	0.66 (0.31, 1.78)	
	Stroke hemisphere (*n* = 38)		Contralateral hemisphere (*n* = 38)
Average Oxy‐Hb (µM mm)		0.44 (−4.68, 17.82)	−0.02 (−4.84, 16.25)
Oxy‐Hb PSD ((µM mm)^2^/Hz)	HF	0.87 (0.29, 5.39)	2.41 (0.47, 12.80)
	LF	1.06 (0.46, 9.11)	3.12 (1.21, 14.84)
	VLF	2.41 (0.46, 15.02)	9.59 (3.32, 27.05)
Coherence	HF	0.36 (0.22, 0.62)	0.33 (0.22, 0.59)
	LF	0.55 (0.31, 0.82)	0.46 (0.26, 0.78)
	VLF	0.48 (0.24, 0.75)	0.38 (0.20, 0.73)
Normalized gain (%/%)	HF	0.71 (0.16, 1.74)	0.66 (0.31, 1.93)
	LF	1.16 (0.41, 3.62)	1.38 (0.64, 3.12)
	VLF	1.60 (0.84, 5.35)	2.38 (1.37, 3.47)
Gain (mm mM/mmHg) × 10^8^	HF	4.87 (3.64, 10.98)	5.50 (4.08, 10.25)
	LF	8.97 (5.82, 20.30)	13.29 (6.10, 25.09)
	VLF	17.29 (9.12, 30.61)	25.05 (11.33, 33.37)
Phase shift (mean, degrees)	HF	23.6 (57.0)	14.1 (57.4)
	LF	39.3 (22.1)	40.4 (20.4)
	VLF	17.2 (22.7)	11.5 (30.3)

^a^ During 5‐min time segment immediately after recanalization. Due to the steady state nature of time segments reported values in relatively representative of mean ABP during most of the procedure.

^b^ During 2‐h time segment or as close as possible to 2‐h post GA.

^c^ Significantly different mean ABP between all timepoints, *P *< 0.001. No significant side‐to‐side differences.

Abbreviations: ABP: arterial blood pressure; $ET_{CO_{2}}$: end‐tidal CO_2_; HR, heart rate; Oxy‐Hb: oxygenated haemoglobin; PSD: power spectral density; $S_{pO_{2}}$, peripheral haemoglobin saturation.

**FIGURE 2:**
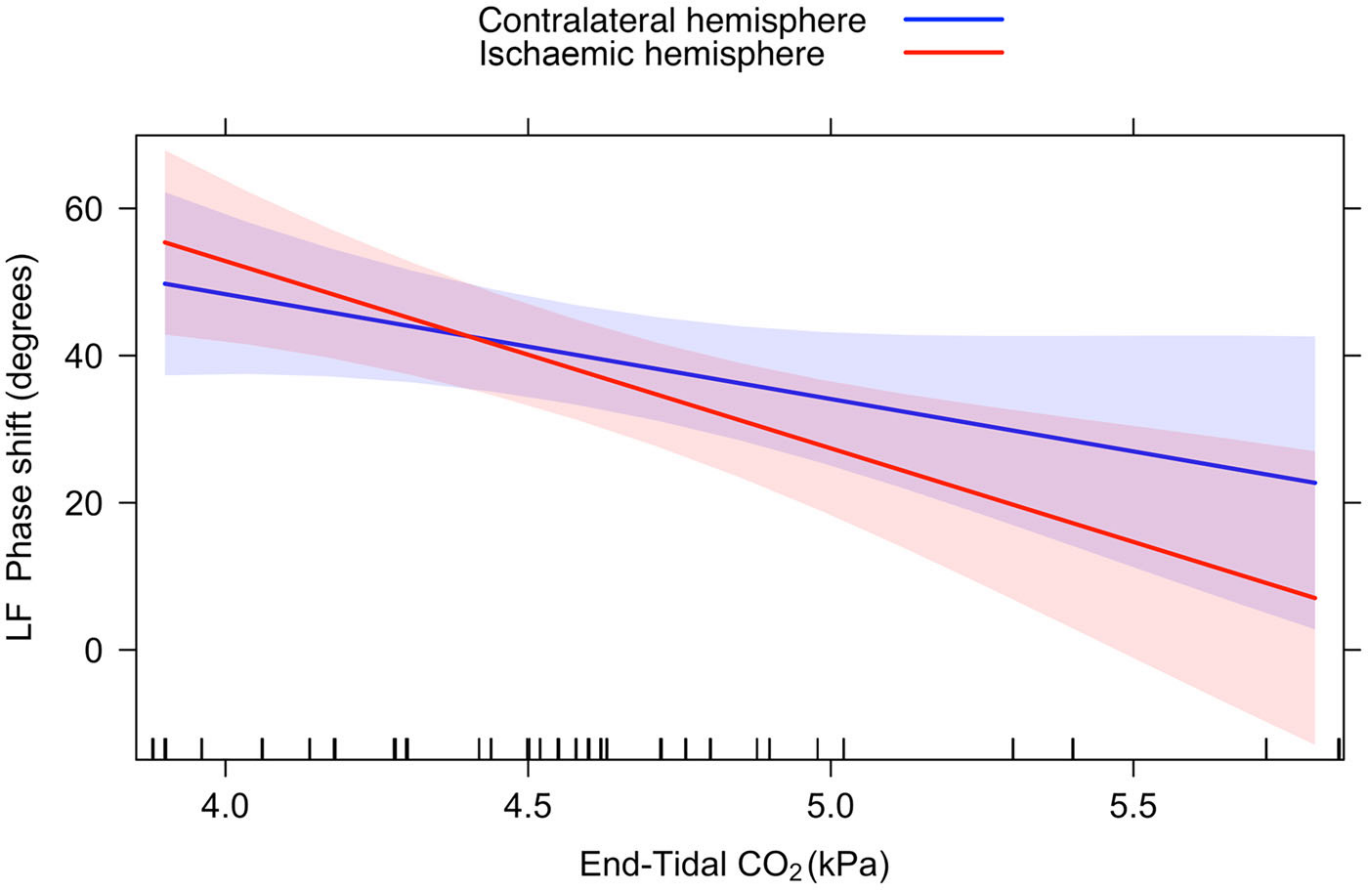
Interaction between $ET_{CO_{2}}$ and hemisphere on LF phase shift with accounting for patient age immediately after recanalization (*n* = 38). The slope of the ischaemic hemisphere is greater than in the contralateral hemisphere.

Accounting for patient age in linear mixed‐effects model showed an interaction with hemisphere (*P* = 0.006). Thus, LF phase shift increased with increasing age in the ischaemic, but not the contralateral, hemisphere. $ET_{CO_{2}}$ also interacted with hemisphere (*P* = 0.015, Figure 2), so that LF phase shift decreased with increasing $ET_{CO_{2}}$ in the ischaemic hemisphere (β: −26.7; 95% CI: −40.8, −12.6; *P *< 0.001) but not in the contralateral hemisphere (β: −14.6; 95% CI: −29.7, 0.5; *P* = 0.058) indicating increased sensitivity to CO_2_. $ET_{CO_{2}}$ decreased linearly with age (−0.01 kPA per year; 95% CI: −0.02, −0.01; *P *< 0.001); combining both $ET_{CO_{2}}$ and age in the same model resulted in preserved hemisphere interaction with $ET_{CO_{2}}$ (*P* = 0.012) but with no certainty concerning hemisphere interaction with age (*P* = 0.050).

LF phase shift of ABP‐OxyHb was independent of stroke aetiology, carotid or intracranial stenosis ≥ 50%, favourable ASPECTS/PC‐ASPECTS before EVT (≥8), occluded territory, NIHSS before EVT, IVT administered before EVT, recanalization success, time from last‐known‐well to recanalization, treatment with antihypertensive medication, average ABP, and all 90‐day outcomes including NIHSS, mRS, independence and mortality (Table 5). We found no interaction between any of the fixed effects and hemisphere.

**TABLE 5:** Linear mixed‐effect models of LF ABP‐OxyHb phase shift immediately after recanalization.

Fixed effect^a^	Hemisphere × fixed effect interaction	Intercept	Fixed effect estimate	Hemisphere estimate *Index: CH*	SS estimate	Model fit
Hemispher*e* *Index: CH*	—	39.1 (CI: 30.7, 47.4)	—	−1.9 (CI: −6.2, 2.4, *T*(34) = −0.91, *P* = 0.368)	0.0 (CI: −0.1, 0.2, *T*(34) = 0.61, *P* = 0.544)	Marginal *R* ^2^: 0.01 Conditional *R* ^2^: 0.83 AIC: 614.6
90‐day mRS (continuous)	*F*(1,33) = 2.04, *P* = 0.163	39.3 (CI: 24.8, 53.8)	−0.1 (CI: −4.0, 4.0, *T*(35) = −0.04, *P* = 0.966)	−1.9 (CI: −6.2, 2.4, *T*(34) = −0.91, *P* = 0.368)	0.0 (CI: −0.1, 0.2, *T*(34) = 0.62, *P* = 0.540)	Marginal *R* ^2^: 0.01 Conditional *R* ^2^: 0.83 AIC: 613.5
90‐day independence (mRS < 3) *Index: Independent*	*F*(1,33) = 0.02, *P* = 0.880	39.6 (CI: 28.4, 50.8)	Dependent: −1.1 (CI: −15.1, 13.0, *T*(35) = −0.15, *P* = 0.878)	−1.9 (CI: −6.2, 2.4, *T*(34) = −0.92, *P* = 0.366)	0.0 (CI: −0.1, 0.2, *T*(34) = −0.64, *P* = 0.530)	Marginal *R* ^2^: 0.01 Conditional *R* ^2^: 0.83 AIC: 610.9
90‐day mortality *Index: Alive*	*F*(1,33) = 1.33, *P* = 0.257	38.2 (CI: 29.0, 47.4)	4.3 (CI: −14.5, 23.1, *T*(35) = 0.46, *P* = 0.648)	−2.0 (CI: −6.2, 2.3, *T*(34) = −0.93, *P* = 0.360)	0.0 (CI: −0.1, 0.2, *T*(34) = −0.68, *P* = 0.502)	Marginal *R* ^2^: 0.01 Conditional *R* ^2^: 0.83 AIC: 610.9
Trichotomized baseline NIHSS *Index: mild (0‐10)*	*F*(2,32) = 0.89, *P* = 0.421	37.5 (CI: 22.5, 52.5)	Moderate (11–19): 2.4 (CI: −15.6, 20.3, *T*(34) = 0.27, *P* = 0.788) Severe (>19): 1.7 (CI: −16.8, 20.1, *T*(34) = 0.19, *P* = 0.854)	−1.9 (CI: −6.2, 2.4, *T*(34) = −0.92, *P* = 0.36)	0.0 (CI: −0.1, 0.2, *T*(34) = 0.62, *P* = 0.540)	Marginal *R* ^2^: 0.01 Conditional *R* ^2^: 0.84 AIC: 606.6
Baseline NIHSS (continuous)	*F*(1,33) = 3.51, *P* = 0.070	37.4 (CI: 19.4, 55.4)	0.1 (CI: −1.3, 1.4, *T*(35) = 0.21, *P* = 0.833)	−1.9 (CI: −6.2, 2.4, *T*(34) = −0.92, *P* = 0.367)	0.0 (CI: −0.1, 0.2, *T*(34) = 0.62, *P* = 0.540)	Marginal *R* ^2^: 0.01 Conditional *R* ^2^: 0.83 AIC: 616.1
90‐day categorized NIHSS *Index: Mild (0‐1)*	*F*(3,31) = 2.16, *P* = 0.113	42.5 (CI: 29.8, 55.1)	Moderate (2–5): −9.8 (CI: ‐26.7, 7.0, *T*(33) = −1.19, *P* = 0.244) Severe (>5): −2.0 (CI: ‐23.0, 19.1, T(33) = −0.19, *P* = 0.851) Dead: 0.0 (CI: −20.9, 20.9, *T*(33) = 0.00, *P* = 0.999)	−1.9 (CI: −6.2, 2.4, *T*(34) = −0.91, *P* = 0.370)	0.0 (CI: −0.1, 0.2, *T*(34) = 0.63, *P* = 0.533)	Marginal *R* ^2^: 0.05 Conditional *R* ^2^: 0.84 AIC: 600.2
CCS *Index: LAA*	*F*(2,29) = 1.06, *P* = 0.360	42.0 (CI: 30.0, 54.1)	CE: −3.4 (CI: −19.3, 12.6, *T*(31) = −0.43, *P* = 0.669) Other: too few observations. Undetermined: −9.7 (CI: −30.2, 10.8, *T*(31) = −0.97, *P* = 0.340)	0.4 (CI: −3.5, 4.3, *T*(31) = 0.20, *P *= 0.845)	0.0 (CI: −0.1, 0.1, *T*(31) = 0.22, *P* = 0.827)	Marginal *R* ^2^: 0.03 Conditional *R* ^2^: 0.88 AIC: 544.3
Carotid or intracranial stenosis (≥50%) *Index: no stenosis*	*F*(1,32) = 0.10, *P* = 0.749	39.0 (CI: 30.4, 47.6)	Stenosis: 0.4 (CI: −7.8, 8.7, *T*(33) = 0.11, *P* = 0.914)	−2.0 (CI: −6.6, 2.6, *T*(33) = −0.88, *P* = 0.384)	0.0 (CI: −0.1, 0.2, *T*(33) = 0.61, *P* = 0.549)	Marginal *R* ^2^: 0.01 Conditional *R* ^2^: 0.83 AIC: 611.9
Favorable ASPECTS before EVT *Index: Favourable*	*F*(1,33) = 0.24, *P* = 0.630	38.0 (CI: 28.2, 47.8)	Unfavourable: 3.1 (CI: −12.0, 18.2, *T*(35) = 0.41, *P* = 0.681)	−1.9 (CI: −6.2, 2.3, *T*(34) = −0.92, *P* = 0.362)	0.0 (CI: −0.1, 0.2, *T*(34) = 0.66, *P* = 0.513)	Marginal *R* ^2^: 0.01 Conditional *R* ^2^: 0.83 AIC: 610.6
Occluded territory *Index: ACA territory including ICA occlusions*	*F*(2,32) = 0.13, *P* = 0.876	40.4 (CI: 27.0, 53.8)	Posterior circulation: −4.5 (CI: −30.2, 21.3, *T*(34) = −0.35, *P* = 0.727) MCA territory only: −1.9 (CI: −17.6, 13.9, *T*(34) = −0.24, *P* = 0.812)	−2.0 (CI: −6.3, 2.3, *T*(34) = −0.93, *P* = 0.360)	0.0 (CI: −0.1, 0.2, T(34) = 0.69, *P* = 0.494)	Marginal *R* ^2^: 0.01 Conditional *R* ^2^: 0.84 AIC: 605.9
Age (continuous)	*F*(1,33) = 8.64, *P* = 0.006 Age × IH: 0.4 (CI: 0.1, 0.7, *T*(33) = 2.94, *P* = 0.006)	37.3 (CI: 0.1, 74.5)	0.0 (CI: −0.5, 0.5, *T*(35) = 0.06, *P* = 0.951)	−30.1 (CI: −50.0, −10.2, *T*(33) = −3.08, *P* = 0.004)	0.1 (CI: −0.1, 0.2, *T*(33) = 0.95, *P* = 0.351)	Marginal *R* ^2^: 0.05 Conditional *R* ^2^: 0.87 AIC: 613.1
Recanalization *Index: Successful*	*F*(1,33) = 0.00, *P* = 0.990	44.6 (CI: 27.3, 61.9)	Unsuccesful: −6.8 (CI: −25.4, 11.8, *T*(35) = −0.74, *P* = 0.464)	−2.0 (CI: −6.2, 2.3, *T*(34) = −0.93, *P* = 0.359)	0.0 (CI: −0.1, 0.2, *T*(34) = 0.69, *P* = 0.496)	Marginal *R* ^2^: 0.02 Conditional *R* ^2^: 0.83 AIC: 609.8
IVT *Index: No IVT*	*F*(1,33) = 1.23, *P* = 0.276	35.2 (CI: 23.9, 46.5)	IVT: 7.0 (CI: −6.7, 20.7, *T*(35) = 1.04, *P* = 0.306)	−2.0 (CI: −6.2, 2.3, *T*(34) = −0.93, *P* = 0.357)	0.0 (CI: −0.1, 0.2, *T*(34) = 0.74, *P* = 0.462)	Marginal *R* ^2^: 0.03 Conditional *R* ^2^: 0.84 AIC: 609.9
Time from LKW to recanalization (continuous, h)	*F*(1,34) = 0.16, *P* = 0.693	40.7 (CI: 31.4, 50.1)	−0.3 (CI: −0.9, 0.4, *T*(35) = −0.84, *P* = 0.409)	−2.0 (CI: −6.3, 2.3, *T*(34) = −0.96, *P* = 0.343)	0.1 (CI: −0.1, 0.2, *T*(34) = 0.83, *P* = 0.413)	Marginal *R* ^2^: 0.02 Conditional *R* ^2^: 0.83 AIC: 616.3
Treatment with antihypertensive medication Index: No antihypertensive	*F*(1,33) = 0.05, *P* = 0.834	37.4 (CI: 24.7, 50.2)	2.5 (CI: −12.2, 17.2, *T*(35) = 0.34, *P* = 0.733)	−1.9 (CI: −6.2, 2.4, *T*(34) = −0.91, *P* = 0.370)	0.0 (CI: −0.1, 0.2, *T*(34) = 0.58, *P* = 0.563)	Marginal *R* ^2^: 0.01 Conditional *R* ^2^: 0.83 AIC: 610.7
Mean ABP (continuous, mmHg)	*F*(1,33) = 1.39, *P* = 0.247	67.9 (CI: 9.4, 126.4)	−0.4 (−1.1, 0.4, *T*(35) = −1.01, *P* = 0.319)	−2.0 (CI: −6.2, 2.4, *T*(34) = −0.91, *P* = 0.368)	0.0 (CI: −0.1, 0.2, *T*(34) = 0.61, *P* = 0.543)	Marginal *R* ^2^: 0.03 Conditional *R* ^2^: 0.83 AIC: 615.8
Average $ET_{CO_{2}}$ (continuous, kPA)	*F*(1,32) = 6.59, *P* = 0.015 $ET_{CO_{2}}$ × IH: −11.2 (CI: ‐20.1, −2.3, *T*(32) = −2.57, *P* = 0.015)	102.8 (CI: 35.6, 170.0)	−13.8 (CI: ‐28.3, 0.6, *T*(34) = −1.94, *P* = 0.060)	49.3 (CI: 8.4, 90.3, *T*(32) = 2.45, *P* = 0.020)	0.0 (CI: −0.1, 0.1, *T*(32) = 0.30, *P* = 0.767)	Marginal *R* ^2^: 0.19 Conditional *R* ^2^: 0.85 AIC: 578.5
Age and $ET_{CO_{2}}$	$ET_{CO_{2}}$ × hemisphere: *F*(1,30) = 7.11, *P* = 0.012 Age × hemisphere: *F*(1,30) = 4.18, *P* = 0.050 $ET_{CO_{2}}$ × age: *F*(1,32) = 0.43, *P* = 0.516	107.2 (CI: 15.5, 198.8)	$ET_{CO_{2}}$: −14.2 (CI: ‐30.0, 1.5, T(33) = −1.84, *P* = 0.075) Age: −0.0 (CI: −0.5, 0.5, *T*(33) = −0.15, *P* = 0.883)	49.3 (8.4, 90.2, *T*(32) = 2.45, *P* = 0.020)	0.0 (CI: −0.1, 0.1, *T*(32) = 0.31, *P* = 0.758)	Marginal *R* ^2^: 0.19 Conditional *R* ^2^: 0.85 AIC: 581.5

^a^ Subjects were applied as random effect and hemisphere as fixed effect in combination with other fixed effects. ACA, anterior cerebral artery; AIC, Akaike information criterion; ASPECTS, Alberta Stroke Program Early CT Score; CCS, Causative Classification System for Ischemic Stroke; CE, cardiac emboli sources; CH, contralateral hemisphere; $ET_{CO_{2}}$, End‐tidal CO_2_; ICA, internal carotid artery; IH, ischaemic hemisphere; IVT, intravenous thrombolysis; LAA, large artery atherosclerosis; LKW, last‐known‐well; MCA, middle cerebral artery; mRS, Modified Rankin Scale; NIHSS, National Institutes of Health Stroke Scale; SS, short‐separation channel TFA regressor.

TFA results based on the complete sequential subset are presented in Table 6. Heart rate and ABP increased after GA at the 2‐h time segment. Contrary to measurements during EVT, normalized gain showed interhemispheric difference in all frequency ranges at the 2‐h segment. Non‐normalized gain increased numerically after recanalization and significantly at the 2‐h segment in the contralateral hemisphere. Our data showed no other complete sequential changes.

**TABLE 6:** Complete sequential analysis of average vitals and TFA results.

		PRE (IQR) (*n* = 11)		POST (IQR) (*n* = 11)		2‐h (IQR) (*n* = 11)	
Examination time^a^		4.4 (3.4, 7.0)		5.6 (4.5, 7.9)		6.5 (6.0, 9.2)	
HR (bpm, mean)		63.9 (16.2)		63.7 (7.3)		**75.1 (20.3)** ^e^	
$S_{pO_{2}}$ (%)		99.6 (97.5, 100)		100 (99.2, 100)		99.6 (96.9, 100)	
$ET_{CO_{2}}$ (kPA)		4.46 (0.41)		4.48 (0.48)		N/A	
ABP (mmHg)		79.6 (73.5, 82.1)		77.0 (75.2, 86.1)		**97.0 (88.0, 105.5)** ^d^ **^,^** ^e^	
ABP PSD (mmHg^2^/Hz)	HF	2.0 (0.9, 8.7)		1.7 (1.3, 3.8)		9.8 (5.8, 12.6)	
	LF	1.1 (0.4, 5.6)		1.1 (0.4, 1.9)		7.0 (4.9, 15.1)	
	VLF	2.6 (0.5, 7.1)		0.4 (0.3, 0.9)		5.0 (3.4, 6.1)	
Hemisphere		Ischaemic	Contralateral	Ischaemic	Contralateral	Ischaemic	Contralateral
Oxy‐Hb (µM*mm)		−2.6 (−17.6, 18.6)	9.1 (−4.7, 11.6)	12.5 (−0.2, 38.4)	−0.0 (−15.5, 23.1)	0.7 (−0.1, 64.7)	−0.1 (−18.7, 0.5)
Oxy‐Hb PSD ((µM*mm)^2^/Hz)	HF	1.0 (0.4, 2.5)	1.5 (0.7, 4.4)	0.3 (0.2, 4.0)	0.8 (0.4, 2.7)	0.1 (0.1, 0.2)	0.3 (0.2, 0.7)
	LF	2.3 (0.2, 6.7)	7.2 (1.9, 28.3)	0.6 (0.3, 3.5)	2.4 (1.3, 3.2)	0.5 (0.3, 2.6)	1.1 (0.7, 8.0)
	VLF	3.4 (1.1, 6.6)	7.8 (3.8, 12.3)	1.6 (0.2, 3.3)	8.5 (5.6, 14.0)	0.8 (0.4, 9.3)	1.9 (1.0, 20.3)
Coherence	HF	0.5 (0.3, 0.7)	0.5 (0.3, 0.7)	0.3 (0.3, 0.6)	0.4 (0.2, 0.7)	0.4 (0.3, 0.6)	0.4 (0.3, 0.6)
	LF	0.7 (0.3, 0.9)	0.8 (0.3, 0.9)	0.5 (0.3, 0.9)	0.5 (0.2, 0.9)	0.6 (0.5, 0.7)	0.6 (0.4, 0.7)
	VLF	0.6 (0.2, 0.8)	0.6 (0.1, 0.8)	0.6 (0.2, 0.8)	0.4 (0.1, 0.8)	0.5 (0.3, 0.7)	0.4 (0.3, 0.6)
Normalized gain (%/%)	HF	0.5 (0.3, 1.0)	0.6 (0.5, 0.7)	0.3 (0.1, 1.1)	0.5 (0.3, 1.1)	**0.3 (0.1, 0.8)** ^b^	**0.7 (0.4, 1.6)** ^b^
	LF	1.0 (0.5, 2.1)	1.3 (0.9, 1.8)	1.4 (0.2, 3.3)	1.4 (0.6, 8.6)	**0.7 (0.2, 2.2)** ^b^	**2.9 (1.1, 3.8)** ^b^
	VLF	1.0 (0.7, 2.3)	1.5 (0.9, 1.6)	2.4 (0.5, 4.1)	1.6 (1.2, 8.1)	**0.7 (0.2, 4.9)** ^b^	**6.6 (1.3, 7.1)** ^b^
Gain (mm*mM/mmHg) *10^8^	HF	8.3 (3.5, 13.2)	4.0 (2.1, 14.6)	9.1 (4.0, 13.0)	5.5 (3.5, 12.0)	8.3 (6.4, 13.8)	5.9 (4.5, 7.6)
	LF	14.8 (5.5, 18.8)	6.3 (2.9, 15.4)^‡^	8.2 (6.4, 30.2)	15.1 (10.3, 29.1)	22.3 (13.3, 41.6)	11.2 (5.9, 22.8)^e^
	VLF	22.5 (13.4, 28.4)	9.5 (6.3, 18.1)^‡^	15.9 (9.5, 38.7)	33.4 (25.0, 34.5)	38.3 (13.6, 55.7)	13.5 (10.5, 32.5)^e^
Phase shift (mean, degrees)	HF	45.1 (74.7)	20.2 (33.2)	20.4 (69.8)	16.8 (66.2)	32.6 (72.2)	31.1 (67.8)
	LF	34.7 (21.8)	29.6 (14.8)	32.7 (25.7)	34.6 (25.9)	32.6 (23.3)	33.7 (27.9)
	VLF	23.2 (31.7)	9.4 (14.2)	10.4 (11.1)	22.9 (33.3)	19.3 (34.8)	11.6 (28.7)

^a^ From Last‐known‐well (h).

^b^ Significant side difference, adjusted *P* < 0.05.

^c^ Difference between PRE and POST segments, adjusted *P* < 0.05.

^d^ Difference between POST and 2‐h segments, *P* < 0.05.

^e^ Difference between PRE and 2‐h, adjusted *P* < 0.05. ABP, arterial blood pressure; $ET_{CO_{2}}$, end‐tidal CO_2_; HR, heart rate; IQR, interquartile range; Oxy‐Hb, oxygenated haemoglobin; PSD, power spectral density; $S_{pO_{2}}$, peripheral haemoglobin saturation.

**FIGURE 3:**
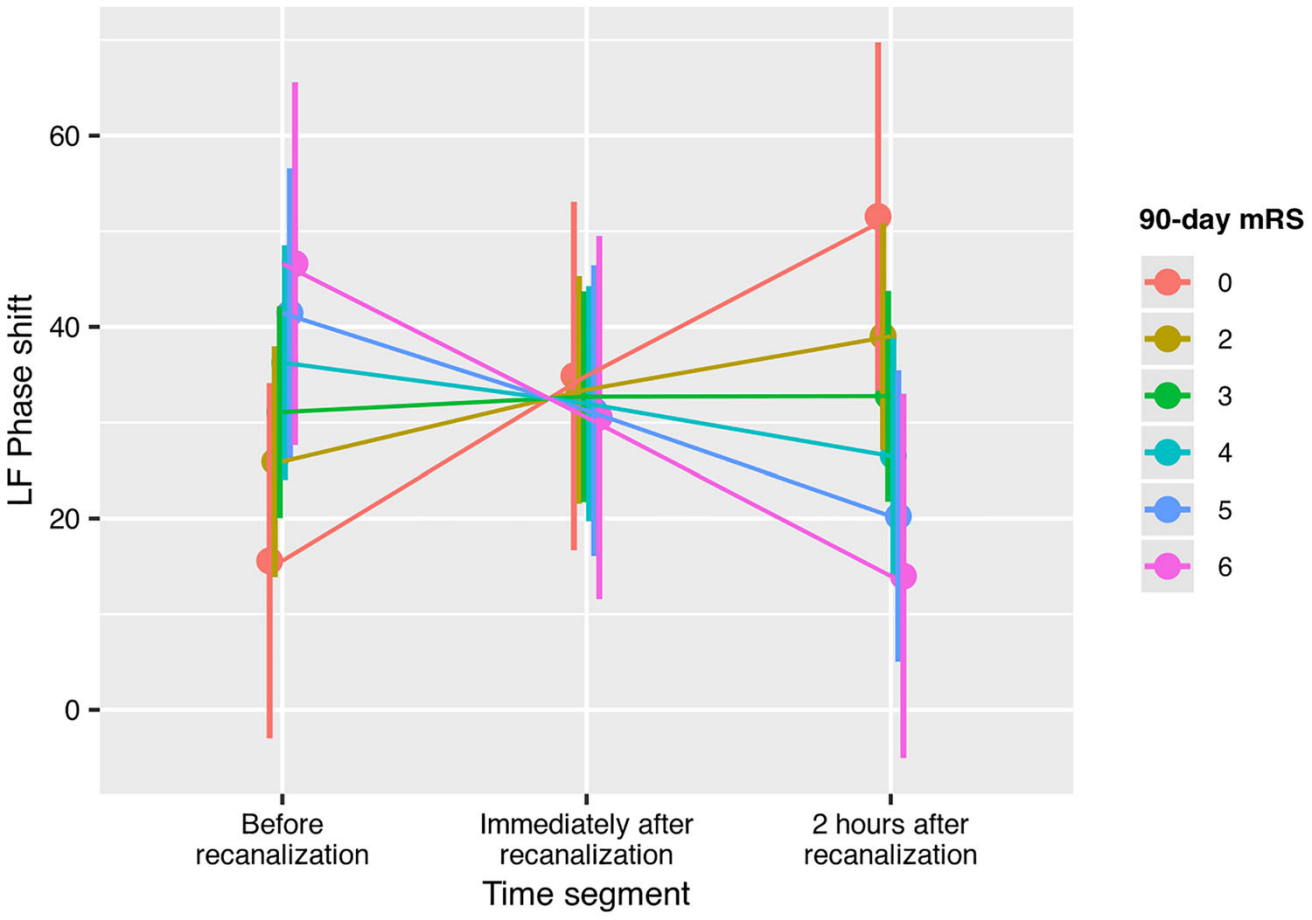
Interaction effect between 90‐day Modified Rankin Scale and time segment on LF phase shift in the complete sequential subset (*n* = 11). Patients with low mRS had progressive increase in phase shift whereas increasing mRS gradually inverted the progression.

**FIGURE 4:**
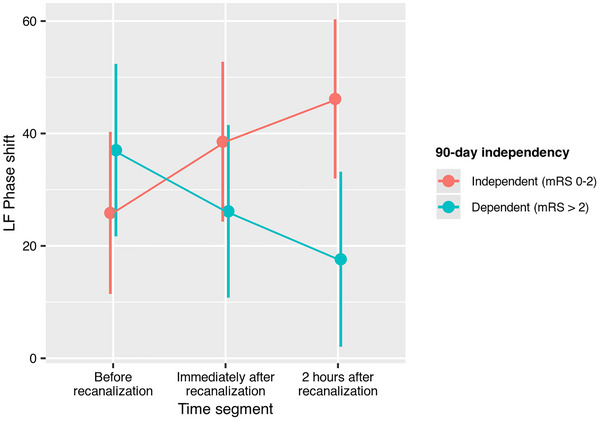
Interaction effect between 90‐day independence (mRS 0–2) and time segment on LF phase shift in the complete sequential subset (*n* = 11). Independent patients exhibited progressive increase in phase shift while the inverse progression was seen in patients with dependency.

**FIGURE 5:**
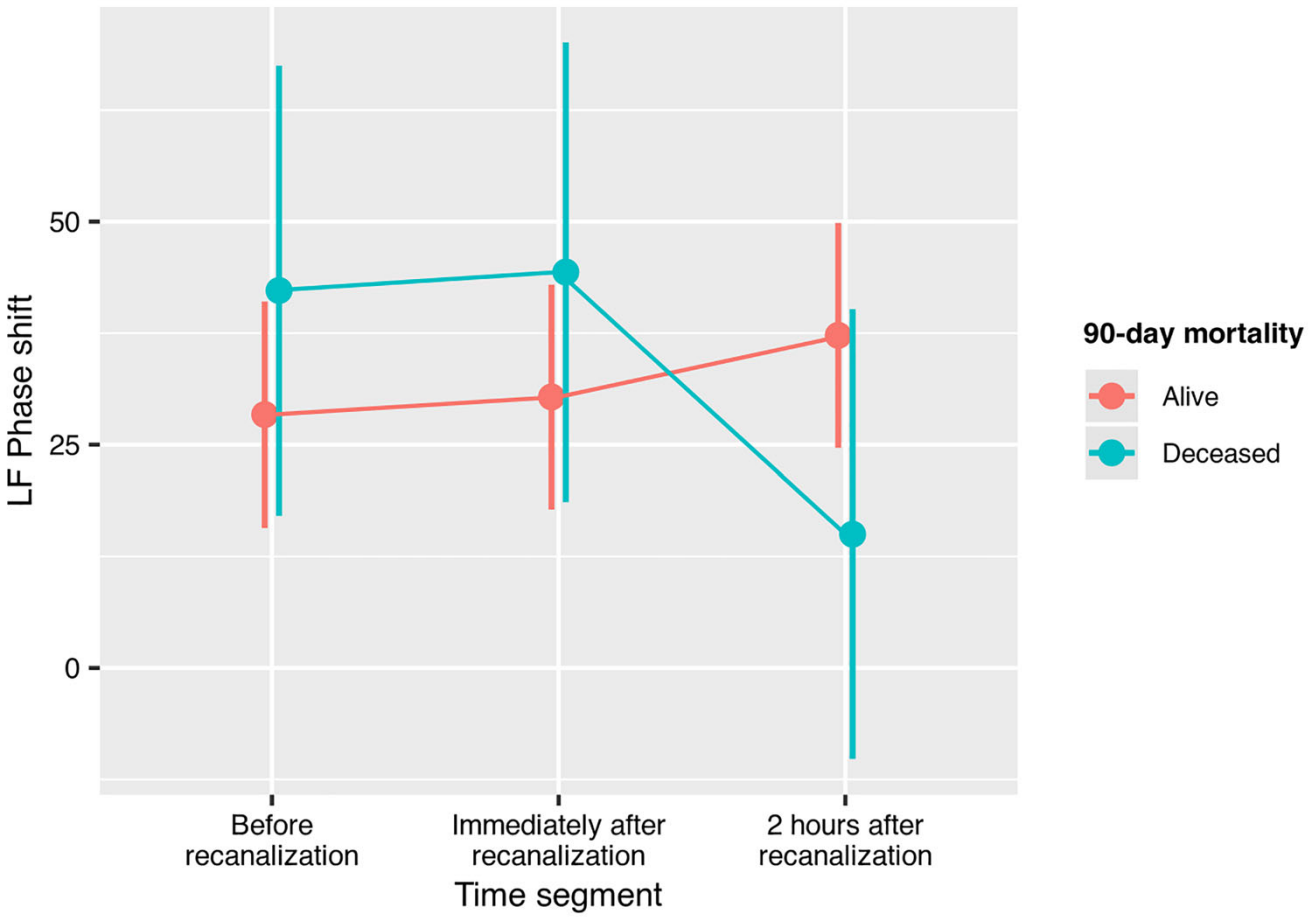
Interaction effect between 90‐day mortality and time segment on LF phase shift in the complete sequential subset (*n* = 11). Phase shift increased between recanalization and 2‐h segment in patients alive at 90‐day follow‐up while the inverse is seen in deceased patients.

Complete sequential mixed‐effect models of phase shift (Table 7) showed that time segment interacted with 90‐day functional outcome measured as categorical mRS (*P* = 0.011, Figure 3), independence (*P* = 0.030, Figure 4) and mortality (*P* = 0.019, Figure 5). Thus, in patients with better 90‐day mRS, LF phase shift was lower before recanalization and increased immediately after recanalization and at the 2‐h segment, while phase shift decreased over time in patients with higher 90‐day mRS. Similar profiles were observed when dichotomizing mRS to independence or mortality. The trend was equal in both hemispheres as there was no interaction between time segment, hemisphere, and 90‐day outcome.

There was no effect of age, stroke aetiology, stenosis ≥50%, baseline NIHSS, time from last‐known‐well to recanalization, nor average ABP in the complete sequential models. We had insufficient sampling to investigate recanalization success, favourable ASPECTS/PC‐ASPECTS before EVT (≥8), $ET_{CO_{2}}$, antihypertensive treatment, and 90‐day NIHSS. No other interaction effects were observed between fixed effects and time segment or hemisphere.

**TABLE 7:** Linear mixed‐effect models of LF ABP‐OxyHb phase shift in complete sequential analysis.

Fixed effect^a^	Time segment × fixed effect interaction	Time segment × hemisphere × fixed effect interaction	Intercept	Fixed effect estimate	Time segment estimate *Index: PRE*	Hemisphere estimate *Index: CH*	SS estimate	Model fit
Hemisphere *Index: CH*	—	*F*(2,49) = 0.30, *P* = 0.746	34.7 (CI: 22.3, 47.2)	—	POST: 2.1 (−10.4, 14.6, *T*(51) = 0.34, *P* = 0.736) 2‐h: 2.6 (CI: −10.0, 15.2, *T*(51) = 0.41, *P* = 0.681)	1.4 (CI: −8.8, 11.6, *T*(51) = 0.28, *P* = 0.784)	−0.1 (−0.2, 0.0, *T*(51) = −1.50, *P* = 0.139)	Marginal *R* ^2^: 0.03 Conditional *R* ^2^: 0.22 AIC: 588.4
90‐day mRS (continuous)	*F*(2,44) = 8.87, *P *< 0.001 POST × 90‐day mRS: −5.9 (CI: −11.4, −0.4, *T*(49) = −2.17, *P* = 0.035) 2‐h × 90‐day mRS: −11.4 (CI: −16.8, −6.0, *T*(49) = −4.26, *P *< 0.001)	*F*(2,44) = 1.13, *P* = 0.332	19.7 (CI: 1.2, 38.6)	5.2 (CI: −0.7, 11.0, *T*(9) = 2.01, *P* = 0.076)	POST: 19.3 (CI: −0.1, 38.7, *T*(49) = 2.00, *P* = 0.051) 2‐h: 36.0 (CI: 16.7, 55.2, *T*(49) = 3.75, *P *< 0.001)	1.4 (CI: −7.5, 10.4, *T*(49) = 0.33, *P* = 0.746)	−0.1 (CI: −0.2, 0.0, *T*(49) = −1.75, *P* = 0.087)	Marginal *R* ^2^: 0.20 Conditional *R* ^2^: 0.43 AIC: 567.6
90‐day independence (mRS < 3) *Index*: *Independent*	*F*(2,44) = 5.93, *P *= 0.005 POST × 90‐day dependent: ‐23.6 (CI: ‐46.3, −0.8, *T*(49) = −2.08, *P* = 0.043) 2‐h × 90‐day dependent: ‐39.7 (CI: ‐62.4, −16.9, *T*(49) = −3.50, *P* = 0.001)	*F*(2,44) = 0.56, *P* = 0.575	29.0 (CI: 14.4, 43.7)	Dependent: 11.2 (CI: −12.1, 34.4, *T*(9) = 1.09, *P* = 0.305)	POST: 12.7 (CI: −2.7, 28.1, *T*(49) = 1.65, *P* = 0.105) 2‐h: 20.3 (CI: 4.8, 35.7, *T*(49) = 2.64, *P* = 0.011)	1.2 (CI: −8.0, 10.5, *T*(49) = 0.27, *P* = 0.789)	−0.1 (CI: −0.2, 0.0, *T*(49) = −1.25, *P* = 0.219)	Marginal *R* ^2^: 0.20 Conditional *R* ^2^: 0.38 AIC: 562.3
90‐day mortality *Index: Alive*	*F*(2,44) = 3.43, *P* = 0.041 POST × 90‐day fatality: 0.1 (CI: −31.2, 31.4, *T*(49) = 0.01, *P* = 0.995) 2‐h × 90‐day fatal outcome: ‐36.2 (CI: ‐66.8, −5.5, *T*(49) = −2.36, *P* = 0.022)	*F*(2,44) = 0.30,*P* = 0.739	31.7 (CI: 18.2, 45.1)	Deceased: 13.9 (CI: −17.7, 45.5, *T*(9) = 1.00, *P* = 0.345)	POST: 2.0 (CI: −11.2, 15.2, *T*(49) = 0.30, *P* = 0.765) 2‐h: 8.9 (CI: −4.4, 22.2, *T*(49) = 1.34, *P* = 0.186)	1.3 (CI: −8.4, 11.0, *T*(49) = 0.26, *P* = 0.793)	−0.1 (CI: −0.2, 0.0, *T*(49) = −1.24, *P* = 0.220)	Marginal *R* ^2^: 01.2 Conditional *R* ^2^: 0.33 AIC: 622.1
Trichotomizedbaseline NIHSS *Index: mild (0‐10)*	*F*(2,34) = 0.12, *P* = 0.888	*F*(2,34) = 0.50,*P* = 0.610	26.7 (CI: 8.3, 45.1)	Moderate (11‐20): 3.3 (CI: ‐20.9, 27.4, *T*(7) = 0.32, *P* = 0.757) Severe (> 20): too few observations	POST: 2.8 (CI: −10.6, 16.3, *T*(41) = 0.43, *P* = 0.671) 2‐h: 8.7 (CI: −4.8, 22.2, *T*(41) = 1.30, *P* = 0.200)	1.6 (CI: −9.3, 12.6, *T*(41) = 0.30, *P* = 0.765)	−0.0 (−0.2, 0.1, *T*(41) = −0.72, *P* = 0.476)	Marginal *R* ^2^: 0.03 Conditional *R* ^2^: 0.33 AIC: 472.1
CCS *Index: LAA*	*F*(2,29) = 0.18, *P* = 0.833	*F*(2,29) = 0.36, *P* = 0.670	35.1 (CI: 17.3, 52.8)	CE: −8.2 (CI: ‐36.0, 19.5, *T*(6) = −0.73, *P* = 0.495) Other and Undetermined: Too few observations	POST: −1.8 (CI: −16.1, 12.5, *T*(36) = −0.25, *P* = 0.801) 2‐h: 4.4 (CI: −9.9, 18.7, *T*(36) = 0.63, *P* = 0.535)	2.3 (CI: −9.4, 13.9, *T*(36) = 0.40, *P* = 0.695)	−0.0 (−0.2, 0.1, *T*(36) = −0.61, *P* = 0.545)	Marginal *R* ^2^: 0.05 Conditional *R* ^2^: 0.35 AIC: 417.1
Carotid or intracranial stenosis (≥ 50%) *Index: No stenosis*	*F*(2,43) = 1.79, *P* = 0.179	*F*(2,43) = 0.69, *P* = 0.505	32.6 (CI: 19.8, 45.3)	Stenosis: 9.5 (CI: −5.0, 23.9, *T*(48) = 1.31, *P* = 0.195)	POST: 2.2 (CI: −10.2, 14.6, *T*(50) = 0.36, *P* = 0.724) 2‐h: 2.8 (CI: −9.8, 15.4, *T*(50) = 0.45, *P* = 0.653)	−1.9 (CI: −13.3, 9.4, *T*(50) = −0.34, *P* = 0.733)	−0.1 (−0.2, 0.0, *T*(48) = −1.71, *P* = 0.094)	Marginal *R* ^2^: 0.07 Conditional *R* ^2^: 0.24 AIC: 582.9
Age (continuous)	*F*(2,44) = 0.57, *P* = 0.570	*F*(2,44) = 0.49, *P* = 0.615	28.6 (CI: −31.1, 88.2)	0.1 (CI: −0.8, 1.0, *T*(9) = 0.21, *P* = 0.839)	POST: 2.1 (CI: −10.4, 14.6, *T*(51) = 0.34, *P* = 0.738) 2‐h: 2.6 (CI: −10.1, 15.2, *T*(51) = 0.41, *P* = 0.686)	1.4 (CI: −8.8, 11.6, *T*(51) = 0.27, *P* = 0.787)	−0.1 (CI: −0.2, 0.0, *T*(51) = −1.45, *P* = 0.153)	Marginal *R* ^2^: 0.03 Conditional *R* ^2^: 0.24 AIC: 590.4
Time from LKW to recanalization (continuous, h)	*F*(2,44) = 0.44, *P* = 0.649	*F*(2,44) = 0.15, *P* = 0.859	34.1 (CI: 14.7, 53.5)	0.1 (CI: −2.5, 2.7, *T*(9) = 0.07, *P* = 0.944)	POST: 2.1 (CI: −10.4, 14.6, *T*(51) = 0.34, *P* = 0.737) 2‐h: 2.6 (CI: −10.1, 15.2, *T*(51) = 0.41, *P* = 0.686)	1.4 (CI: −8.8, 11.6, *T*(51) = 0.27, *P* = 0.786)	−0.1 (CI: −0.2, 0.0, *T*(51) = −1.43, *P* = 0.158)	Marginal *R* ^2^: 0.03 Conditional *R* ^2^: 0.24 AIC: 588.3
Average ABP (continuous, mmHg)	*F*(2,43) = 1.69, *P* = 0.197	*F*(2,43) = 0.02, *P* = 0.980	−1.0 (CI: ‐40.6, 38.6)	0.4 (CI: −0.0, 0.9, *T*(50) = 1.90, *P* = 0.063)	POST: 2.1 (CI: −10.1, 14.2, *T*(50) = 0.34, *P* = 0.734) 2‐h: −5.1 (CI: ‐19.9, 9.7, *T*(50) = −0.69, *P* = 0.491)	1.4 (CI: −8.5, 11.4, *T*(50) = 0.28, *P* = 0.778)	−0.1 (CI: −0.2, 0.0, *T*(50) = −1.55, *P* = 0.127)	Marginal *R* ^2^: 0.10 Conditional *R* ^2^: 0.28 AIC: 587.9

^a^ Subjects were applied as random effect and hemisphere as fixed effect in combination with other fixed effects. AIC, Akaike information criterion; ASPECTS, Alberta Stroke Program Early CT Score; CCS, Causative Classification System for Ischemic Stroke; CE, cardiac emboli sources; CH, Contralateral hemisphere; LAA, large artery atherosclerosis; LKW, last‐known‐well; mRS, Modified Rankin Scale; NIHSS, National Institutes of Health Stroke Scale; SS, short‐separation channel TFA regressor.

## 4 DISCUSSION

The current study displays the feasibility of examining dCA during EVT with NIRS in the conventional TFA setup between ABP and cerebral circulation (*V*_MCA_, OxyHb). We observed no difference in dCA between hemispheres at any point in time during or after EVT. However, immediately after recanalization, dCA as measured by phase shift displayed an increased sensitivity to $ET_{CO_{2}}$ changes in the ischaemic hemisphere compared to the contralateral hemisphere, suggesting increased susceptibility to perturbation of cerebral blood flow with changes in ventilation.

In the complete sequential subset, we found an interaction between time segments and 90‐day functional outcome including mortality. Thus, LF phase shift increased over time in patients with good outcome, suggesting progressive stabilization of dCA, and decreased in patients with poor outcome, suggesting progressive impairment of dCA.

The focus of this study was the phase shift between ABP and OxyHb in the LF range. Gain was previously examined by interhemispheric TFA using NIRS as both input and output and produced more stable results with less variation (Heiberg et al., 2025). Some of the observed variation of gain in the current study could be explained by differences in measurement modality, metabolic or oxygen extraction changes affecting OxyHb, and blood flow contributions from multiple sources in watershed borderzones or from collateral circulation measured by NIRS. Unlike gain, there is no unilateral measure of phase shift when performing interhemispheric TFA. Likewise, ABP and OxyHb oscillations in the VLF range examine another interesting aspect of dCA but require longer time segments with steady‐state data (Panerai et al., 2023), which was not as available in this clinical setting.

LFOs in cerebral circulation are usually examined by TCD, which has consistently shown a phase lead of *V*_MCA_ compared to ABP in both healthy subjects and stroke patients (Intharakham et al., 2019; Panerai et al., 2023). However, stroke patients with impaired dCA have a reduced phase lead of *V*_MCA_ indicating a more passive transfer of LFOs from the systemic perfusion into the cerebral circulation (Diehl et al., 1995). Conversely, OxyHb LFOs in healthy subjects follow those in ABP, which has been interpreted as transit time from large vessels to the cerebral microvasculature (Müller & Österreich, 2019; Phillip et al., 2012; Reinhard et al., 2006). Spontaneous and respiratory enhanced LFOs have been estimated at 15–24° in rather narrow frequency ranges (0.09−0.11 and 0.06−0.12 Hz). Parameter setting including frequency ranges explain most of the variance between TFA results from corresponding cohorts and equal settings would be preferable in comparisons between cohorts (Meel‐van den Abeelen et al., 2014). Müller & Österreich (2019) applied a wider‐ranging frequency spectrum (0.07−0.15 Hz) that better resembles the white paper guidelines and the current study (0.07−0.2 Hz) and reported a higher phase lead of 55 degrees.

The effect of impaired dCA on ABP‐OxyHb is not yet established. Presuming the relationship between *V*_MCA_ and OxyHb remains the same; LF phase shift of ABP‐OxyHb would be expected to increase with dCA impairment. However, disruption of the *V*_MCA_‐OxyHb phase shift was shown despite unaltered ABP‐OxyHb phase shift in carotid stenosis patients compared to healthy subjects (Reinhard et al., 2006). Some extent of phase shift would be expected between ABP and OxyHb with intact dCA, and decreasing phase shift could also be an indication of reduced autoregulatory mechanisms following the reasoning by Diehl et al. (1995). The understanding of decreasing LF ABP‐OxyHb phase shift with impaired dCA would seem to fit well when comparing our results to findings by Müller and Österreich (2019). The observed effect of decreasing phase shift with increasing $ET_{CO_{2}}$ also supports this interpretation of dCA impairment as the influence of hypercapnia has been reported in numerous studies (Johnson et al., 2025; Minhas et al., 2018; Panerai et al., 1999). Conversely, we cannot discard the possibility of decreased phase shift serving as an adaptive response to increase oxygen supply in acute ischaemic conditions by decreasing the temporal delay. Such a response would seem harmful as decreased phase shift in the ischaemic hemisphere has consistently been associated with worse functional outcome (Beishon et al., 2024; Nogueira et al., 2022). If the decreased phase shift represents an adaptive response, this would also extend into the non‐ischaemic areas (i.e., as there was no difference in phase shift between isolated MCA occlusions and ICA occlusions) and the contralateral hemisphere.

*V*_MCA_ estimates the indirect upstream effect of dCA whereas OxyHb measures cortical haemoglobin concentration mostly in the arterial vascular compartment and could offer a more direct dCA assessment. However, as OxyHb from the capillary bed and venous compartment cannot be separated from the arterial compartment and ABP‐OxyHb, LF phase shift could therefore be affected by microvascular shunts as well as the venous outflow as well (Müller et al., 2020). Although microvascular and macrovascular dCA correlates well (Phillip et al., 2012; Reinhard et al., 2006) and have even produced similar values when accounting for high‐frequency features (Elting et al., 2020; Mol et al., 2021), the relation between *V*_MCA_, ABP and OxyHb require further investigations, which should include the venous outflow to better guide the interpretation of NIRS‐based dCA assessment. Hybrid measurements with diffuse correlation spectroscopy (NIRS‐DCS) could provide microvascular blood flow estimation and facilitate interpretation (Favilla et al., 2023).

The interpretation of dCA based on OxyHb is complicated as OxyHb is not a direct measure of cerebral blood flow. OxyHb is associated with relative cerebral blood flow (Gomez et al., 2022; Polinder‐Bos et al., 2020; Reinhard et al., 2006) but could also be affected by levels of blood gases and ventilation status (Payne et al., 2011), loss of blood, metabolic demand, oxygen extraction and capillary recruitment (Suppan et al., 2022) as well as inflammatory changes related to cerebral ischaemia (De Meyer et al., 2022; Eleveld et al., 2021). While dCA estimation based on NIRS could be affected by the factors mentioned above, blood flow velocity could also be affected by some of the same factors. The prevailing dCA estimation based on blood flow velocity is therefore also affected by some of the same uncertainties. We controlled for some of the factors in the selection of time segments (steady state of mechanical ventilation, OxyHb and vitals), while changes occurring outside the LF frequency range should not impact the result of the individual time segment TFA. However, such factors could change substantially between time segments in the complete sequential analysis. Metabolic demand and capillary recruitment could change due to recanalization. Further changes could be seen after termination of GA, which would also include changes to ventilation, blood gases and inflammatory responses. Our interpretation of dCA in the complete sequential analysis should therefore be read with a fair amount of uncertainty.

The possible dCA effect from anaesthesia and vasopressor medications have previously been described with propofol doses exceeding 200 µg/kg/min (Claassen et al., 2021) and sevoflurane levels above 1.5 minimum alveolar concentration (Slupe & Kirsch, 2018), but such doses were not applied during EVT in the current study. The two patients in our cohort receiving sevoflurane had similar phase shifts compared to the remaining patients (data not shown). Adding remifentanil to propofol does not affect dCA (Engelhard et al., 2001). Neither noradrenaline (Meng, Sun, Zhao, Rasmussen, et al., 2024) nor phenylephrine (Meng, Sun, Zhao, Meng, et al., 2024) affects dCA in healthy subjects. However, the effect of the applied anaesthetics, opioids and vasopressors has not been examined in acute ischaemic stroke patients. In the current cohort we did not observe any difference between any dCA measures when such medications were discontinued after GA.

Mean ABP also showed changes between time segments that could theoretically impact dCA. Hypertension before EVT was expected due to compensatory hypertension, high levels of arousal or anxiety (Bath et al., 2018), and temporary non‐compliance of antihypertensives due to the index stroke. Inducing GA caused a lower mean ABP that increased again after EVT. Prominent hypotension (Tzeng et al., 2010) and malignant hypertension (Immink et al., 2004) have previously been shown to affect dCA but not at the mean ABP in the current cohort. Our analysis did not show any effect of mean ABP and should therefore be robust against such changes.

Previous studies of dCA in stroke patients with large‐vessel occlusions have been based on TCD and only after recanalization therapy. Consistent findings across studies show impaired dCA in the ischaemic hemisphere compared to healthy subjects, while most studies also show some extent of dCA impairment in the contralateral hemisphere (Tian et al., 2020). Several TFA studies found a reduction in ABP‐*V*_MCA_ phase lead bilaterally but less pronounced in the contralateral hemisphere (Castro, Serrador, et al., 2017; Ran et al., 2024; Sheriff et al., 2020; Tian et al., 2020). As the current study showed no interhemispheric difference in dCA at earlier stages, we suspect that partial remission of dCA impairment after recanalization occurs earlier in the contralateral hemisphere after recanalization, which could be consistent with our previous findings showing increased interhemispheric phase shift after 24 h (Heiberg et al., 2025).

We observed an increasing phase shift and dCA change with age in the ischaemic hemisphere, but this was largely explained by decreasing levels of $ET_{CO_{2}}$ with patient age. Elderly subjects are expected to have less compensatory reserves due to increasing levels of endothelial dysfunction, cerebral small‐vessel disease and arterial stiffness (Beishon et al., 2021). Increasing dCA with patient age would therefore have been quite surprising and contradictory to previous studies (Beishon et al., 2021; Phillip et al., 2012), which include conventional ABP‐*V*_MCA_ setups (Madureira et al., 2017; Maxwell et al., 2022) that found no effect of ageing.

Meanwhile, hypercapnia would be expected to increase cerebral blood flow (CBF) and influence dCA in both hemispheres based on previous studies of healthy subjects (Johnson et al., 2025; Minhas et al., 2018; Panerai et al., 1999). The increased sensitivity to $ET_{CO_{2}}$ in the ischaemic hemispheres could be an indication of dCA impairment as CBF under such circumstances would be more dependent on other regulation mechanisms. Hypercapnia has shown some association to improved outcome (Scudellari et al., 2023) and the direct effect to increase CBF could be more impactful on outcome than the effect on dCA. Examining the individual dCA differences across varying levels of $ET_{CO_{2}}$ in future studies would solidify our finding. Other unexposed confounders or effect modifiers including intracranial pressure (Czosnyka et al., 2005) could also be involved.

Numerous studies have associated LF phase shift in the ischaemic hemisphere of acute stroke patients to long‐term functional outcome (Beishon et al., 2024; Nogueira et al., 2022). We did not observe this association immediately after recanalization, but the complete sequential analysis offers a potential explanation for this discrepancy. Patients with good long‐term outcome (low mRS) had lower phase shift before recanalization which increased immediately after recanalization and 2‐h post‐procedure, while the trend gradually reverted with increasing mRS. Although the complete sequential analysis was based on a limited number of patients, the trend was remarkably consistent across mRS categories. All mRS categories intersected immediately after recanalization making it impossible to associate long‐term mRS with LF phase shift at that time segment. Categorizing mRS to functional independence or mortality showed a similar trend although with different intersection points. Improvement in dCA would be expected in patient with better functional outcome (Nogueira et al., 2021) and could suggest that the observed increase in ABP‐OxyHb LF phase shift indicates dCA restoration. Changes in dCA during and shortly after EVT could have clinical implications that need further investigations. Promising results have been shown when personalizing blood pressure limits with dCA after EVT (Petersen et al., 2020; Zhang et al., 2024), and a randomized trial is ongoing (ClinicalTrials.gov: NCT05670028). Similar studies before and during EVT could be performed using NIRS.

### 4.1 Limitations

The current study was performed in a challenging clinical setting which merits some limitations. EVT is a hyperacute and time‐sensitive treatment that leaves no opportunity for unnecessary delays. Thus, we experienced significant recruitment (100 of 484 patients examined with NIRS) challenges as investigators were required to arrive at the angiosuite before or simultaneously with participants to enable setup before EVT was initiated. Such challenges could be minimized with a larger team of sub‐investigators including the regular staff. A large proportion of patients examined with NIRS were excluded from analysis (20%) due to missing continuous ABP monitoring during EVT as further attempts to place the arterial line would have delayed the procedure. Future studies could benefit from consistent use of ABP measurement from the access sheath and subsequent placement of a radial arterial line between recanalization and termination of GA to monitor ABP after the procedure. Further, many patients had to be excluded because of missing steady state periods, especially from the complete sequential subset. The window for identifying time segments after induction of GA and stabilization after before recanalization was quite narrow and often not available in patients with very rapid recanalization attempts occurring especially when attaining steady state was difficult (e.g., due to complex comorbidity or high sensitivity to anaesthetics or vasopressors). In time segments after GA, the availability was limited by clinical requirement for prolonged GA, noisy data during clinical follow‐up imaging and postoperative agitation due to confusion, anxiety or aphasia, or non‐steady data (e.g. labile blood pressure, frequent administration of acute antihypertensives). Some of these challenges could be minimized in future studies with a larger team of sub‐investigators including the regular staff better covering non‐office hours. The relatively small sample size limited our possibilities for constructing more complex statistical models with multiple clinical variables.

Another substantial limitation of this study is that we have not examined healthy controls subjects, which constrains our interpretation of possible dCA impairment. Due to ethical concerns, we refrained from pursuing dCA examinations under GA. Performing dCA examinations on healthy subjects during non‐vascular surgery (e.g. orthopaedic) was considered, but invasive ABP is often not included as standard‐of‐care monitoring in such settings (Nguyen & Bora, 2025). Further, such patients could perhaps be affected by pain sensation and blood loss, and patients developing postoperative cognitive dysfunction or delirium would need to be excluded (Chuan et al., 2019; Longhitano et al., 2021). While dCA examinations performed during wakefulness would also have been a possibility, an arterial line catheter as used here for ABP measurement would be unpleasant for healthy participants. Alternatively, dCA based on non‐invasive and invasive ABP measurements can be compared directly when applying certain corrections (Petersen et al., 2014) but not without considerable reliability concerns (Olsen et al., 2022; Panerai et al., 2023). Applying *V*_MCA_ measurements after EVT would also have been interesting and could possibly confirm the presented results.

Using NIRS is also restricted by other limitations. Spatial depth is limited to the superficial cortex impeding dCA investigations of deeper cortical and subcortical regions (Ferrari & Quaresima, 2012). While regional dCA differences in the superficial cortex can be investigated using multi‐channel NIRS, we found no evidence of this in the prefrontal cortex during preliminary analysis. Further, no prevalent method has been able to filter extracerebral signals from intracerebral signals creating uncertainty concerning the extent of extracerebral contamination (Eleveld et al., 2023). To account for this issue, we performed a second TFA based on short‐distance channels examining the extracerebral tissues and applied the results as a regressor in the mixed‐effect models. Exploring the exact intracerebral sensitivity using this method is beyond the scope of this study.

In conclusion, NIRS can be applied during EVT to examine dCA. In our study there was no difference between dCA of the ischaemic and contralateral hemisphere at any time point. However, the sensitivity of dCA to $ET_{CO_{2}}$ was increased in the ischaemic hemisphere. We found dCA changes during and 2 h after EVT with increasing phase shift in patients with good long‐term functional outcome and decreasing phase shift in patients with poor outcome. Further investigations are warranted to compare the reported dCA assessment to the conventional approach based on TCD in both healthy subjects and stroke patients. Individualizing blood pressure thresholds during EVT in patients with dCA changes based on NIRS seems plausible and could be beneficial for their clinical outcome.

## AUTHOR CONTRIBUTIONS

Adam Vittrup Heiberg and Helle Klingenberg Iversen conceived and designed the study while Thomas Clement Truelsen, Henrik Gutte Borgwardt, Goetz Benndorf, Christine Sølling, and Henrik Winther Schytz contributed. Adam Vittrup Heiberg acquired most of the data with substantial contributions from Troels Gil Lukassen as well as Thomas Clement Truelsen, Henrik Gutte Borgwardt, Goetz Benndorf, Christine Sølling and Klaus Hansen Adam Vittrup Heiberg performed data processing and analysis with contributions from Troels Gil Lukassen and Helle Klingenberg Iversen All authors participated in the interpretation of results. Adam Vittrup Heiberg drafted the article. All authors revised the article critically and approved the final version. All persons qualifying for authorship was designated as authors and have agreed to be accountable for all aspects of the work and resolve of questions related to the integrity or accuracy of the study.

## CONFLICT OF INTEREST

All authors state that there are no competing interests.

## Data Availability

The processing code is open source available at https://openfnirs.org/software/homer/ for preprocessing and at https://www.car‐net.org/tools for TFA. NIRS data segments can be provided upon reasonable request. Individual patient data are identifiable, and availability requires approval of both centres as well as local ethics committees. All data requests can be made by contacting the corresponding author.
